# The status of interprofessional medical and nursing education in wound care at German higher education institutions

**DOI:** 10.3205/zma001841

**Published:** 2026-04-15

**Authors:** Anna Weiß, Constanze Richters, Raisa Kiriakidou, Anita Hausen, Martin R. Fischer, Matthias Stadler, Matthias J. Witti

**Affiliations:** 1 LMU University Hospital, LMU Munich, Institute of Medical Education, Munich, Germany; 2 Catholic University of Applied Sciences Munich, Munich, Germany

**Keywords:** medical education, interprofessional education, interprofessional collaboration practice, nursing, physicians, wound care, chronic wound

## Abstract

**Objective:**

Chronic wounds pose an increasing challenge to the German healthcare system and require solid knowledge in wound care. Interprofessional education can contribute to improving the quality of care. This study aimed to evaluate the current state of interprofessional wound care education in medical and nursing programs at German higher education institutions.

**Methods:**

Based on the National Competence-Based Learning Objectives Catalog for Medicine (NKLM), expert standards and the Nursing Professions Training and Examination Ordinance (PflAPrV), a questionnaire with four closed and two open items was developed to assess the curricular integration of learning objectives, teaching methods, and interprofessional concepts. The survey was distributed to teaching staff responsible for medical programs (n=40) and primary qualifying nursing programs (n=30) in Germany. The data were analyzed using descriptive statistics and qualitative content analysis.

**Results:**

A total of 77 teaching staff members participated in the survey (58 from medicine and 19 from nursing). The majority stated that they teach learning objectives related to wound care in their courses. This indicates that this topic is broadly integrated into the curriculum, albeit with varying degrees of depth in terms of content and structural design. Interprofessional teaching formats are offered by 21% of nursing programs and 60% of medical programs. Nursing programs are characterized by a practice-oriented, coherent approach, while medical programs show fragmentation across different professions and semesters.

**Conclusion:**

Despite curricular integration, structured interprofessional learning opportunities in wound care are still needed.

## Zusammenfassung

**Zielsetzung:**

Chronische Wunden stellen eine wachsende Herausforderung für das deutsche Gesundheitssystem dar und erfordern fundierte Kenntnisse in der Wundversorgung. Interprofessionelle Lehre kann zur Verbesserung der Versorgung beitragen. Ziel der Studie war es, den Ist-Stand der Lehre zur interprofessionellen Wundversorgung im Medizinstudium und im primärqualifizierenden Pflegestudium in Deutschland zu erheben.

**Methodik:**

Basierend auf dem Nationalen Kompetenzbasierten Lernzielkatalog Medizin (NKLM), den Expertenstandards sowie der Pflegeberufe-Ausbildungs- und -Prüfungsverordnung (PflAPrV) wurde ein Fragebogen mit vier geschlossenen und zwei offenen Items zur curricularen Integration von Lernzielen, Lehrmethoden und interprofessionellen Ansätzen entwickelt. Die Befragung richtete sich an Lehrverantwortliche (LV) der staatlichen Medizinstudiengänge (n=40) und der primärqualifizierenden Pflegestudiengänge (n=30) in Deutschland. Die Auswertung erfolgte deskriptiv-statistisch und qualitativ mittels strukturierender Inhaltsanalyse.

**Ergebnisse:**

Insgesamt nahmen 77 LV (58 Medizin und 19 Pflege) an der Umfrage teil. Die Mehrheit gab an, Lernziele zur Wundversorgung in ihren Lehrveranstaltungen zu vermitteln, was auf eine breite curriculare Verankerung hinweist – allerdings mit unterschiedlicher inhaltlicher Tiefe und struktureller Ausgestaltung. Interprofessionelle Lehrformate bieten 21% der Pflegestudiengänge und 60% der Medizinstudiengänge an. Während das Pflegestudium durch eine praxisorientierte und kohärente Vermittlung geprägt ist, zeigt sich im Medizinstudium eine Fragmentierung über verschiedene Fachrichtungen und Semester hinweg.

**Schlussfolgerung:**

Trotz curricularer Verankerung besteht ein Bedarf an strukturierten, interprofessionellen Lernangeboten zur Wundversorgung.

## 1. Introduction and problem definition

The increase in life expectancy is leading to an aging population, which poses challenges for the healthcare system. Chronic diseases are on the rise, resulting in more hospital treatments [1]. Additionally, advancing age is associated with a higher risk of multimorbidity and the need for care [2]. One of these health challenges are chronic wounds [3]. In Germany, the prevalence of chronic wounds in 2012 was 1.1% of the total population [3]. The most common chronic wounds are pressure ulcers, leg ulcers, and diabetic foot syndrome [4]. Chronic wounds are a significant health burden for patients and cause high costs for the healthcare system [5]. Wound care encompasses all interventions used to treat wounds in order to promote healing and prevent complications [6]. To meet these societal challenges and ensure cost-effective, patient-centered care, innovative interprofessional care solutions that go beyond single-profession formats must be found [7]. Evidence-based wound care requires specific professional skills and career prospects that are anchored in both the medical and nursing fields and reflected in teaching. There is currently a lack of data on the extent and depth of wound care teaching in medical studies and primary qualifying nursing programs in Germany, both in monoprofessional and, in particular, interprofessional formats. It is also unclear if and how interprofessional courses on wound care are anchored in the curricula in Germany and which didactic and methodological concepts are used in medicine and nursing.

### 1.1. Current state of research

The current state of research on wound care in medical education reveals challenges in implementing the learning objectives outlined in the National Competency-Based Learning Objectives Catalog for Medicine (NKLM) [https://nklm.de/zend/menu]. For instance, the section on “wounds and ulcers of the skin and mucous membranes” involves eleven different disciplines, resulting in a fragmented education that does not always provide the necessary depth and coherence [8]. This fragmentation, in terms of time and content, of teaching could be one reason for students' uncertainty and incomplete understanding in the field of wound care [8]. A survey from 2008 found that, on average, only seven hours are spent teaching wound care throughout the entire clinical part of medical studies in Germany [9]. In addition to the compulsory curriculum, elective courses on wound care can be a useful addition to medical studies in order to adequately meet the growing need for chronic wound care [10], [11]. A systematic survey on the didactic implementation of wound care in medical studies is still missing. The Nursing Professions Training and Examination Ordinance (PflAPrV) [12] integrates specific learning content on wound care into the theoretical and practical training of nurses. Nurses must be able to plan and implement evidence-based interventions. These regulations ensure that wound care is a central component of nursing education [12]. International studies show that nursing students often have limited competence in wound care [13], [14]. Additionally, many students report that they did not receive sufficient training in this area during their studies [15]. Previous studies have shown that these deficits can be remedied through targeted educational interventions [16]. In Germany, the majority of wound care is provided by nurses [17], yet it remains unclear how wound care is integrated into the nursing curriculum and what didactic concepts are used to teach it. Wound care is an interprofessional field that involves both nurses and physicians. It requires successful interprofessional collaboration (IPC). The primary goal of IPC is to ensure safe, patient-centered care and achieve the best possible quality of care for patients [18], [19]. The WHO [20] describes IPC as the coordinated collaboration between healthcare professionals from different professional backgrounds to ensure high-quality care in collaboration with patients and their relatives. Interprofessional education (IPE) of future team members is considered a prerequisite for successful IPC [21]. IPE occurs when trainees, students, or professionals from different professions come together to learn with, from, and about each other [20]. The aim of IPE is to optimize collaboration in healthcare and thus improve the quality of care and patient outcomes [22]. To date, we are only aware of one interprofessional course at a German medical university that addresses joint wound care [23]. However, recent studies show that interprofessional education, training, and continuing education reduce medical errors and promote patient safety [24], [25], [26], [27]. Teaching programs, such as lectures, tutorials, and simulation learning, are particularly important in raising students’ awareness of their role in the healthcare team and fostering a shared approach to patient-centered wound care [28]. Especially in the field of wound care, interprofessional teams have been shown to decisively improve treatment outcomes [29], [30].

### 1.2. Research questions

Despite the existence of defined learning objectives in the NKLM [https://nklm.de/zend/menu], PflAPrV [12], and DNQP expert standards [17], [31], it remains unclear to what extent the topic of wound care is covered in mono- and interprofessional teaching in German medical and primary qualifying nursing programs. A systematic assessment of the current status to subsequently develop targeted, needs-oriented and adaptable educational programs for mono- and interprofessional training in wound care is necessary in order to contribute to patient safety in the long term. 

In light of the aforementioned background/context, we ask the following research questions:

- RQ1: To what extent are the contents of mono- and interprofessional wound care represented in the curricula of medical and nursing higher education institutions in Germany?
- RQ2: What didactic concepts and methods are used in mono- and interprofessional courses on the subject of wound care in medicine and nursing?

## 2. Methods

### 2.1. Study design

A quantitative survey was conducted and combined with qualitative, free-text responses. The questionnaire was created on the SoSci Survey platform [32] (see attachment 1 ).

### 2.2. Sample

To obtain the data, all German state medical faculties (n=40) and all nursing schools (n=30) offering primary qualifying courses in nursing in accordance with the Nursing Professions Act were included. In the medical program, teaching staff responsible for the subjects of surgery, dermatology, and internal medicine were included, following the approach used in the study by Werdin et al. [9]. Since nursing education does not have an analogous subject structure to that of medicine, all nursing program teaching staff selected by program coordinators were included.

### 2.3. Questionnaire

Based on the learning objectives of the NKLM [https://nklm.de/zend/menu], the expert standard for the care of people with chronic wounds [31], the expert standard for pressure ulcer prevention [17], and the PflAPrV [12], a questionnaire was developed comprising a total of six items, consisting of four closed and two open questions (see attachment 1 , Questionnaire). The closed questions assessed the extent to which the learning objectives are taught in the courses. In addition, characteristics of the specific programs were recorded using drop-down menus and multiple-choice or multiple-response answers. The open-ended questions enabled the teaching staff to provide qualitative data that offered detailed insights into the teaching concepts and methods used for mono- and interprofessional wound care. The focus was on time-efficient implementation (approx. 5 minutes), using a short test instrument to enable integration into everyday clinical practice.

### 2.4. Data collection

Data collection took place from April to October of 2024. 

In the field of medicine, all faculty deans at state universities were contacted by email. They were asked to give their consent to study participation and to provide the contact details of the teaching staff. The teaching staff were then invited to participate in the survey via email. In the field of nursing, program coordinators were contacted directly by email and asked for their consent and participation in the survey. 

After the faculty deans and program coordinators gave their consent, the contact details of the courses were used to send individual survey invitations by email. These invitations were sent via official channels. To achieve a comprehensive response rate, multiple follow-up requests were made.

### 2.5. Data analysis

The quantitative data (four closed items) were evaluated using Microsoft Excel 2016 descriptive statistics to determine how many universities address the learning objectives for wound care. 

The qualitative data from the two open-ended questions were evaluated using MAXQDA Analytics Pro 2024 (version 24.8.0) according to the structured qualitative content analysis method developed by Kuckartz and Rädiker [33]. Four main categories were developed in the category system, three of which were based on curriculum development according to Schlutz [34]. All material was coded separately for medicine and nursing using the main categories. After coding was complete, a category-based analysis and summary of the main categories were carried out.

### 2.6. Quality criteria

To ensure intersubjectivity, the codes were discussed and jointly reflected upon by the research team. Transparency was ensured through systematic documentation of the development of the category system and the coding process.

### 2.7. Ethics

The study was conducted in accordance with the ethical principles of the Declaration of Helsinki (WMA). All participants were informed about the purpose of the study, the voluntary nature of participation and the confidential handling of their data.

## 3. Results

### 3.1. Sample description

A total of 235 teaching staff members were contacted via email. 94 participants began the survey. A total of 77 complete data sets were obtained, including 58 teaching staff from 20 medical programs and 19 teaching staff from nursing programs (total response rate: 32.77%). The participating medical faculties and nursing schools are located throughout Germany, except in the federal states of Hessen and Bremen.

### 3.2. RQ1

The results show that 19 out of 20 medical faculties teach learning objectives relating to hygiene, diagnostics, pathophysiology, and practical skills in wound care. Seventeen faculties also incorporate non-pharmacological therapy principles. One faculty stated that it does not teach any of the learning objectives. Table 1 (Tab. 1) shows which wound care learning objectives are taught at the surveyed faculties. The table contains the learning objectives, as well as the number and percentage of faculties at which they are taught.

The results show that 18 out of 19 nursing schools teach wound assessment and classification of wounds and the surrounding area. Sixteen schools teach wound treatment and appropriate dressing recommendations, while 15 schools teach evidence-based wound care. Fourteen schools teach skills for caring for chronic wounds, assessing wound care and explaining wound management in an interprofessional team. One school does not teach any of these learning objectives. Table 2 (Tab. 2) shows which wound care learning objectives are taught at the surveyed nursing schools. The table contains the learning objectives, as well as the number and percentage of schools that teach them.

### 3.3. RQ2

Four main deductive categories were coded based on the open-ended responses. Three of these categories relate to three characteristics of curriculum development. These are: learning content, teaching method and timing [34]. A total of 141 text segments from the open-ended responses were assigned to these main categories. Table 3 (Tab. 3) shows all main categories relevant to the evaluation results, as well as the number of coded text segments in each category.

The main category, “learning content”, was assigned when teaching staff commented on their course content. 

Courses on wound care at medical schools cover a wide range of topics and vary in content and focus depending on the program of study. Students acquire basic knowledge in areas such as *“the fundamentals of wound science, physiology [of] wound healing, and signs [of] wound infection” *(HM, Pos. 2). Practical skills such as hygienic dressing changes, wound observation, and wound suturing and care are trained on models and patients. Treatment of acute wounds, including burns, as well as chronic wounds, such as pressure ulcers and diabetic foot syndrome, are central components of medical education.

Courses on wound care at nursing schools cover a wide range of topics and vary in content and focus depending on the institution. Students acquire knowledge about wound healing processes, wound assessment, wound documentation and the requirements of relevant guidelines and* “application of expert standards (skin integrity, pressure ulcer prevention, chronic wounds, discharge management”* (PF, Pos. 63). Practical exercises include bandaging techniques, aseptic and septic dressing changes, wound cleaning, wound dressings and emergency treatment for acute wounds. The management of chronic wounds is taught with consideration of interdisciplinary cooperation and personalized therapy planning. Seminars explore topics such as anatomy, physiology and pathology of the vascular system, as well as other causes of chronic wounds, in greater depth.

The main category “method” was assigned when the teaching staff commented on the method used in the course.

Wound care content is taught in medical school using various teaching formats that depend on the program's location and structure. These formats include lectures (n=10), seminars (n=12), bedside teaching (n=12), block clerkships (n=11) and online units (n=3). They are integral parts of the curriculum and are offered in various disciplines, such as dermatology, surgery and internal medicine. Medical study courses vary in duration and intensity, ranging from one-off sessions to modules lasting several hours and block internships.

In nursing studies, wound care content is taught through a combination of theoretical and practice-oriented teaching methods, which vary depending on the location and structure of the program. Lectures (n=7), seminars (n=11), skills lab exercises (n=10), and real-world practice exercises (n=7) are integral parts of the curriculum. This content is embedded in modules such as scientific and biomedical fundamentals and evidence-based nursing. 

The main category “timing” was assigned when the teaching staff commented on the timing of a course in the degree program. 

The timing of teaching wound care content varies depending on the location and stage of study. Wound care content is rarely taught in the preclinical semesters (especially in the 3^rd^ and 4^th^ semesters), more frequently in the early clinical semesters (5^th^ to 7^th^ semesters) and most frequently in the higher clinical semesters (9^th^ and 10^th^ semesters) and in the practical year.

The timing of wound care content in nursing schools varies depending on the location and course structure. Most often, relevant content is taught in the 2^nd^ and 3^rd^ semesters, while it is much less common in the higher semesters.

The quantitative survey shows that interprofessional courses are taught in 21% of nursing programs and 60% of medical programs.

The main category “IPE” was assigned when the teaching staff commented on the interprofessional orientation of the teaching. 

In medical studies, interprofessional education is evident in the *“involvement of the wound manager in practical exercises”* (HM, Pos. 14) and in joint rounds. 

In nursing studies, interprofessional education takes place through the collaboration *“between nurses and physicians”* (PF Pos. 27).

## 4. Discussion

The survey on the integration of wound care content into medical school curricula shows that the topic is broadly anchored in the curriculum, but with significant differences in the structure and design of teaching. Interprofessional learning formats are rarely offered.

In medical studies, teaching often takes place in modules spread over different semesters. This fragmentation, in terms of both time and content, could explain why medical students are uncertain about wound care [9], making it difficult to develop a comprehensive understanding of the complex wound care process across disciplinary and professional boundaries.

Although relevant learning objectives for wound care are defined in the NKLM [https://nklm.de/zend/menu] and, as the results show, these are also taught broadly, albeit with considerable variation, the question arises as to whether both their number and their depth of content are sufficient to meet the increased demands of chronic wound care. Studies suggest that deepening wound care education and incorporating practical training can be useful additions to medical studies [10], [11]. 

In contrast, nursing studies appear to teach wound care in a more coherent and practical manner. Students acquire both theoretical foundations and specific practical skills. The program is based on the PflAPrV guidelines [12] and the DNQP expert standards [17], [31], suggesting a coherent, action-oriented approach to teaching wound care skills. Furthermore, the importance of thorough wound care education is reflected in clinical practice: in Germany, nurses provide the majority of wound care [17]. One possible reason for this is the abundance of continuing education and training courses tailored specifically to nurses. Professional associations such as the “Initiative Chronische Wunde” [https://www.icwunden.de/] offer standardized qualification programs that contribute to professionalization and quality assurance in wound care. The competence deficits in nursing students in the field of wound care described in the international literature [13], [15] also support the assumption that a purely curricular anchoring does not necessarily lead to confidence in practice. Therefore, supplementary educational courses are a promising approach to addressing existing gaps. For instance, the wound care competence of certified nurses could be enhanced through targeted continuing education programs.

While wound care content is taught in both professions, the interprofessional dimension is often neglected. Although teaching staff from both nursing and medical programs reported integrating IPE elements, the free-text responses indicate that these do not align with the WHO definition of interprofessional education [20]. This suggests that a structured interprofessional teaching concept in wound care is lacking, despite the fact that clinical care clearly demonstrates the benefits of interprofessional teams. Studies show that team collaboration effectively promotes healing and reduces the risk of wound recurrence [29], [30].

### 4.1. Limitations

A key limitation of this study is that not all medical schools or nursing schools participated in the survey, and the responses could not be clearly assigned to individual disciplines. Therefore, it is unclear whether the information was provided by a single member of the teaching staff or if certain disciplines were not represented at all. This limitation may have distorted the results. Nursing vocational education was not taken into account, even though this is where most nurses are trained and where different conditions for IPE may apply in some cases.

## 5. Conclusion

The survey results show a high demand for structured learning opportunities in wound care, a need that universities partially address. Furthermore, the findings reveal that interprofessional education is scarcely implemented despite evidence showing that interprofessional care concepts can improve treatment outcomes. Therefore, expanding interprofessional learning opportunities could further enhance practice-oriented training. Integrating this into the curriculum could specifically promote key competencies among medical and nursing students, better preparing them for collaborative care teams. Future research should focus on the development and evaluation of such educational formats.

## Acknowledgements

The authors would like to thank the faculty deans and program coordinators for their support and the teaching staff for their participation in the study.

## Notes

### Funding

Foundation Stiftung Innovation in der Hochschullehre (Freiraum 2022; FR-153/2023).

### Authors’ ORCIDs

- Anna Weiß: [0009-0006-5187-2740]
- Constanze Richters: [0000-0003-1593-3543]
- Anita Hausen: [0009-0006-3929-4861]
- Martin R. Fischer: [0000-0002-5299-5025]
- Matthias Stadler: [0000-0001-8241-8723]
- Matthias J. Witti: [0000-0002-5758-1948]

## Competing interests

The authors declare that they have no competing interests. 

## Supplementary Material

Questionnaire on interprofessional wound care

## Figures and Tables

**Table 1:**
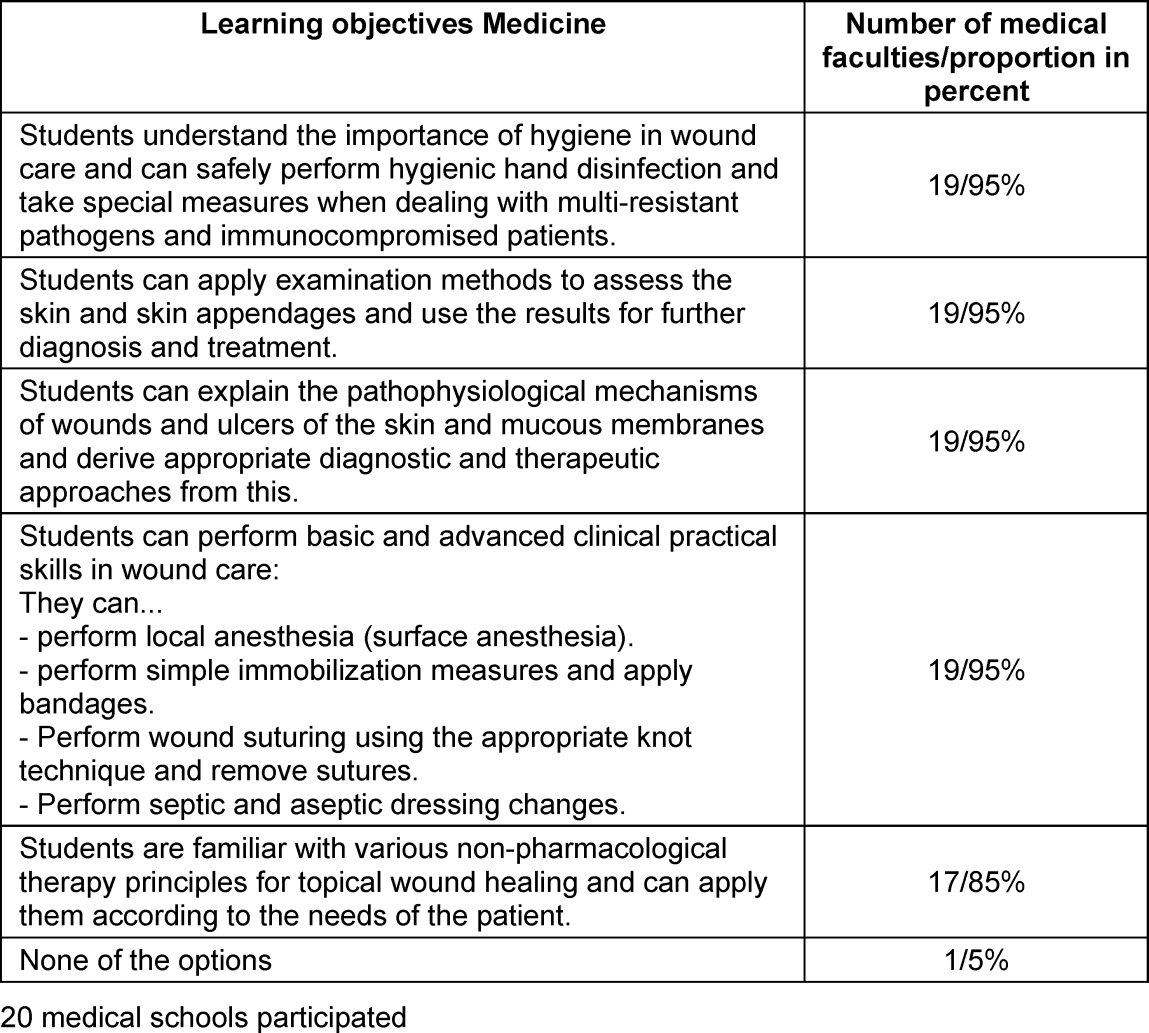
Teaching of learning objectives in the medical degree program

**Table 2:**
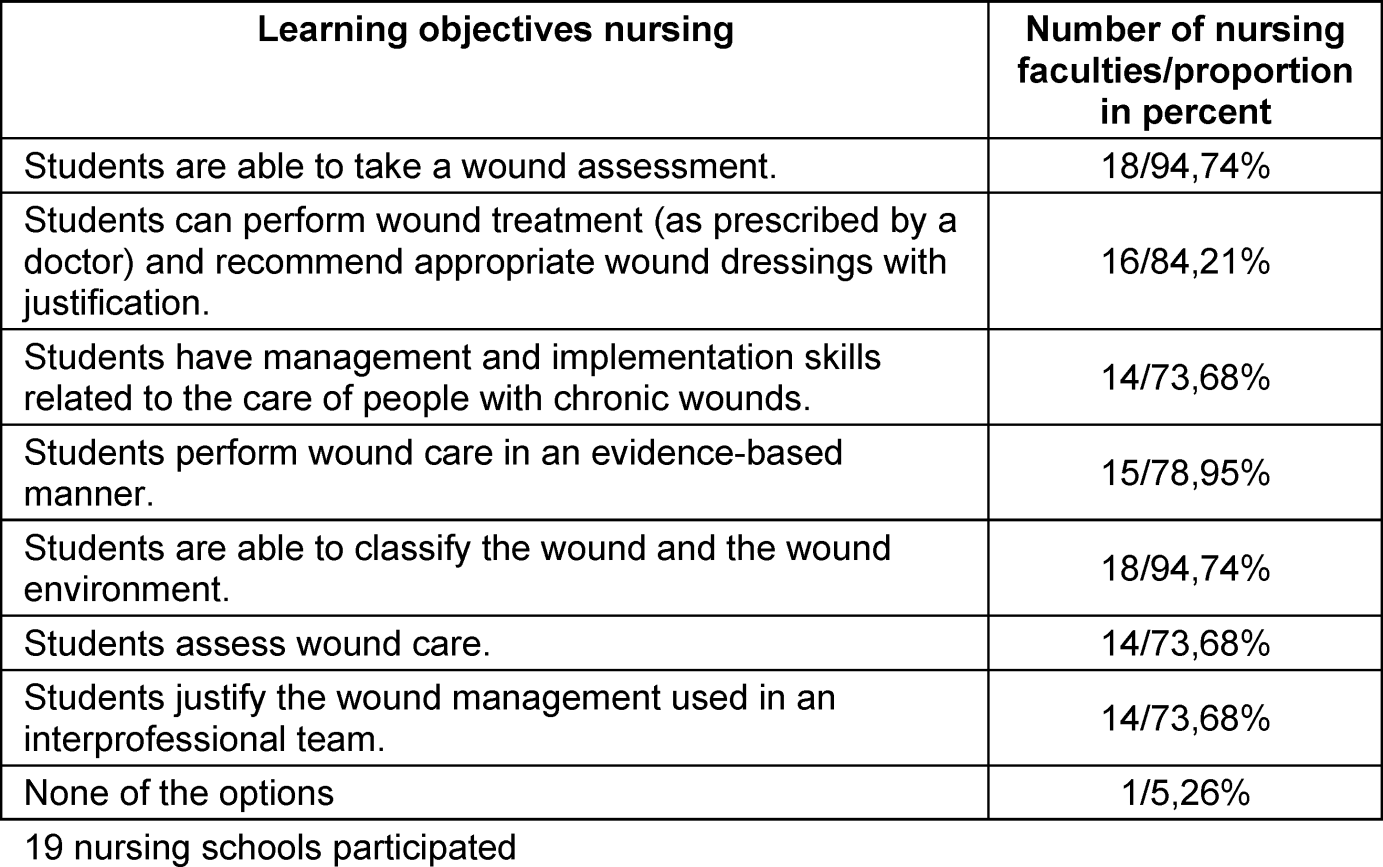
Teaching of learning objectives in the nursing degree program

**Table 3:**
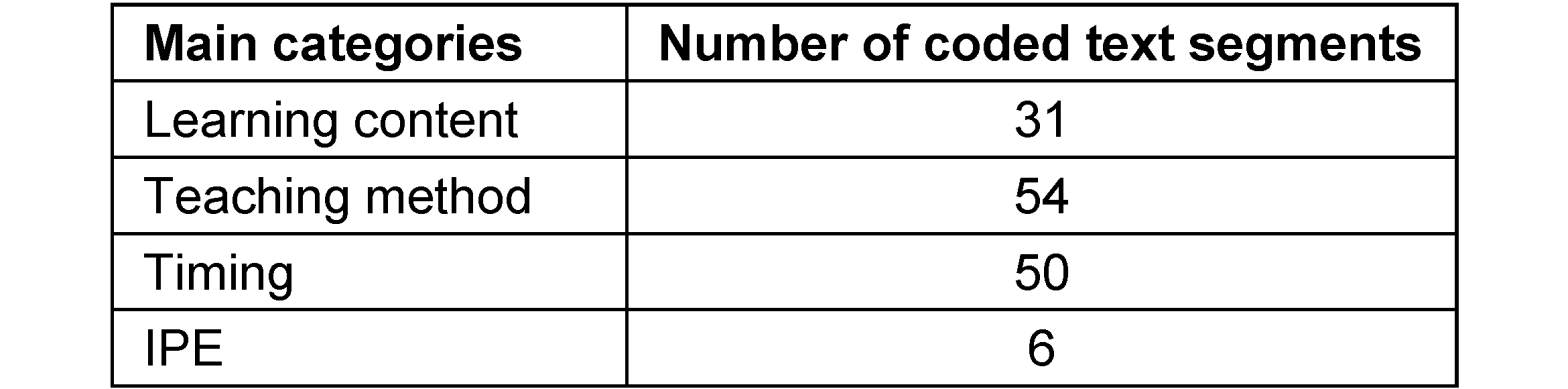
List of relevant main categories

## References

[1] Nowossadeck E (2012). Demografische Alterung und Folgen für das Gesundheitswesen. GBE kompakt.

[2] Robert Koch Institut (2015). Gesundheit in Deutschland. Gesundheitsberichterstattung des Bundes. Gemeinsam getragen von RKI und Destatis.

[3] Heyer K, Herberger K, Protz K, Glaeske G, Augustin M (2016). Epidemiology of chronic wounds in Germany: Analysis of statutory health insurance data. Wound Repair Regen.

[4] Krüger K, Dissemond J, Dissemond J, Kröger K (2019). Chronische Wunden: Diagnostik – Therapie – Versorgung.

[5] Purwins S, Herberger K, Debus ES, Rustenbach SJ, Pelzer P, Rabe E, Schäfer E, Stadler R, Augustin M (2010). Cost-of-illness of chronic leg ulcers in Germany. Int Wound J.

[6] Deutsche Gesellschaft für Wundheilung und Wundbehandlung e.V. S3-Leitlinie Lokaltherapie schwerheilender und/oder chronischer Wunden aufgrund von peripherer arterieller Verschlusskrankheit, Diabetes mellitus oder chronischer venöser Insuffizienz. AWMF-Registernummer: 091-001.

[7] Boersema GC, Smart H, Giaquinto-Cilliers MGC, Mulder M, Weir GR, Bruwer FA, Idensohn PJ, Sander JE, Stavast A, Swart M, Thiart S, Van der Merwe Z (2021). Management of Nonhealable and Maintenance Wounds: A Systematic Integrative Review and Referral Pathway. Adv Skin Wound Care.

[8] Bergendahl L, Werner F, Schmidt A, Ronicke M, Renner R, Erfurt-Berge C (2020). Entwicklung und Evaluation eines interprofessionellen Lehrkonzepts zum modernen Wundmanagement. J Dtsch Dermatol.

[9] Werdin F, Fischer A, Schaller HE, Schönfisch B, Rennekampff HO (2008). Undergraduate education on chronic wound care at German medical schools--the role of surgical disciplines. Handchir Mikrochir Plast Chir.

[10] Kamath P, Agarwal N, Salgado CJ, Kirsner R (2019). Wound healing elective: an opportunity to improve medical education curriculum to better manage the increasing burden of chronic wounds. Dermatol Online J.

[11] Fox JD, Baquerizo Nole KL, Berriman SJ, Kirsner RS (2016). Chronic Wounds: The Need for Greater Emphasis in Medical Schools, Post-graduate Training and Public Health Discussions. Ann Surg.

[12] Bundesministerium für Gesundheit (2018). PflAPrV Pflegeberufe-Ausbildungs- und -Prüfungsverordnung.

[13] Kielo-Viljamaa E, Suhonen R, Ahtiala M, Kolari T, Katajisto J, Salminen L, Stolt M (2021). The development and testing of the C/WoundComp instrument for assessing chronic wound-care competence in student nurses and podiatrists. Int Wound J.

[14] Welsh L (2018). Wound care evidence, knowledge and education amongst nurses: a semi-systematic literature review. Int Wound J.

[15] Kielo E, Salminen L, Suhonen R, Puukka P, Stolt M (2019). Graduating student nurses' and student podiatrists' wound care competence: a cross-sectional study. J Wound Care.

[16] Martinengo L, Yeo NJ, Markandran KD, Olsson M, Kyaw BM, Car LT (2020). Digital health professions education on chronic wound management: A systematic review. Int J Nurs Stud.

[17] Deutsches Netzwerk für Qualitätsentwicklung in der Pflege (2017). Expertenstandard Dekubitusprophylaxe in der Pflege.

[18] Kaap-Fröhlich S, Ulrich G, Wershofen B, Ahles J, Behrend R, Handgraaf M, Herinek D, Mitzkat A, Oberhauser H, Scherer T, Schlicker A, Straub C, Waury Eichler R, Wesselborg B, Witti M, Huber M, Bode SF (2022). Position paper of the GMA Committee Interprofessional Education in the Health Professions - current status and outlook. GMS J Med Educ.

[19] Reeves S, Lewin S, Espin S, Zwarenstein M (2010). Interprofessional Teamwork in Health and Social Care.

[20] World Health Organization (2010). Framework for action on interprofessional education and collaborative practice.

[21] Homeyer S, Hoffmann W, Hingst P, Oppermann R, Dreier-Wolfgramm A (2018). Effects of interprofessional education for medical and nursing students: enablers, barriers and expectations for optimizing future interprofessional collaboration – a qualitative study. BMC Nurs.

[22] Witti MJ, Zottmann JM, Wershofen B, Thistlethwaite JE, Fischer F, Fischer MR (2023). FINCA - a conceptual framework to improve interprofessional collaboration in health education and care. Front Med (Lausanne).

[23] Erfurt-Berge C (2024). Wundversorgung – ein Lehrthema mit Potenzial. Aktuelle Derm.

[24] Cox M, Cuff P, Brandt B, Reeves S, Zierler B (2016). Measuring the impact of interprofessional education on collaborative practice and patient outcomes. J Interprof Care.

[25] Kangas S, Rintala TM, Jaatinen P (2018). An integrative systematic review of interprofessional education on diabetes. J Interprof Care.

[26] Reeves S, Fletcher S, Barr H, Birch I, Boet S, Davies N, McFadyen A, Rivera J, Kitto S (2016). A BEME systematic review of the effects of interprofessional education: BEME Guide No. 39. Med Teach.

[27] Mette M, Christina B, Jutta H, Narciß E (2021). Gaining interprofessional knowledge and interprofessional competence on a training ward. Med Teach.

[28] Parker CN, Johnston S, Theobald KA (2022). Promoting Person-Centered Care for Health Baccalaureate Students: Piloting an Interprofessional Education Approach to Wound Management. Adv Skin Wound Care.

[29] Moore Z, Butcher G, Corbett LQ, McGuiness W, Snyder RJ, van Acker K (2014). Exploring the concept of a team approach to wound care: Managing wounds as a team. J Wound Care.

[30] Heerschap C, Nicholas A, Whitehead M (2018). Wound management: Investigating the interprofessional decision-making process. Int Wound J.

[31] Deutsches Netzwerk für Qualitätsentwicklung in der Pflege (2015). Expertenstandard Pflege von Menschen mit chronischen Wunden.

[32] Leiner DJ (2024). SoSci Survey (Version 3.5.07).

[33] Kuckartz U, Rädiker S (2024). Qualitative Inhaltsanalyse: Methoden, Praxis, Umsetzung mit Software und künstlicher Intelligenz.

[34] Schlutz E (2006). Bildungsdienstleistungen und Angebotsentwicklung.

