# Supplementary material for: The status of interprofessional medical and nursing education in wound care at German higher education institutions
Source: GMS J Med Educ. 2026 Apr 15;43(4):Doc47. doi: 10.3205/zma001841 (PMC13124531; doi:10.3205/zma001841)
Supplement: Questionnaire on interprofessional wound care [file JME-43-47-s-001.pdf]

Attachment 1: Questionnaire on interprofessional wound care

Sociodemographic data:

1. At which university do you work?

- ☐ Albert-Ludwigs-Universität Freiburg
- ☐ Alice Salomon Hochschule Berlin
- ☐ Brandenburgische Technische Universität
- ☐ Carl-von-Ossietzky-Universität Oldenburg
- ☐ Charité Berlin
- ☐ Christian-Albrechts-Universität Kiel
- ☐ Eberhard Karls Universität Tübingen
- ☐ Ernst-Abbe-Hochschule Jena
- ☐ Ernst-Moritz-Arndt-Universität Greifswald
- ☐ Evangelische Hochschule Berlin
- ☐ Evangelische Hochschule Dresden
- ☐ Evangelische Hochschule Ludwigsburg
- ☐ Evangelische Hochschule Nürnberg
- ☐ Fachhochschule Bielefeld
- ☐ Fliedner-Fachhochschule Düsseldorf
- ☐ Friedrich-Alexander-Universität Erlangen-Nürnberg
- ☐ Friedrich-Schiller-Universität Jena
- ☐ Friedrich-Wilhelms-Universität Bonn
- ☐ Freie Universität Berlin
- ☐ Georg-August-Universität Göttingen
- ☐ Heinrich-Heine-Universität Düsseldorf
- ☐ Hochschule Bremen
- ☐ Hochschule Esslingen
- ☐ Hochschule für Angewandte Wissenschaften Hamburg
- ☐ Hochschule für Gesundheit Bochum
- ☐ Hochschule für angewandte Wissenschaften Kempten
- ☐ Hochschule für Wirtschaft und Gesellschaft Ludwigshafen
- ☐ Hochschule Fulda
- ☐ Hochschule München
- ☐ Hochschule Neubrandenburg

- Hochschule Zittau/Görlitz
- Johannes-Gutenberg-Universität Mainz
- Julius-Maximilians-Universität Würzburg
- Justus-Liebig-Universität Gießen
- Katholische Stiftungshochschule München
- Ludwig-Maximilians-Universität München
- Martin-Luther-Universität Halle-Wittenberg
- Medizinische Hochschule Hannover
- Ostbayerische Technische Hochschule Regensburg
- Otto-von-Guericke-Universität Magdeburg
- Pädagogische Hochschule Schwäbisch Gmünd
- Philipps-Universität Marburg
- Rheinische Friedrich-Wilhelms-Universität Bonn
- Ruprecht-Karls-Universität Heidelberg
- Technische Hochschule Deggendorf
- Technische Hochschule Rosenheim
- Technische Universität Dresden
- Technische Universität München
- Universität Augsburg
- Universität Bielefeld
- Universität Duisburg-Essen
- Universität Frankfurt am Main
- Universität Greifswald
- Universität Hamburg
- Universität Köln
- Universität Leipzig
- Universität Lübeck
- Universität Münster
- Universität Regensburg
- Universität Rostock
- Universität Saarlandes
- Universität Trier
- Universität Ulm
- Westfälische Wilhelms-Universität Münster

Question type: Multiple Choice (MC)

2. How many students are enrolled in your degree program?
Number of students:
Question type: Open numerical question

3. For which degree program are you completing this survey?
- Medicine
-Nursing
Question type: Multiple choice (MC)

4 a. Do you teach the learning objectives for wound care from the National Competency-Based Learning Objectives Catalog for Medicine (NKLM) in your degree program? (only for Medicine)

- ☐ Students understand the importance of hygiene in wound care and can safely perform hygienic hand disinfection and take special measures when dealing with multi-resistant pathogens and immunocompromised patients.
- ☐ Students can apply examination methods to assess the skin and skin appendages and use the results for further diagnosis and treatment.
- ☐ Students can explain the pathophysiological mechanisms of wounds and ulcers of the skin and mucous membranes and derive appropriate diagnostic and therapeutic approaches from this.
- ☐ Students can perform basic and advanced clinical practical skills in wound care:
  They can...
  -perform local anesthesia (surface anesthesia).
  -perform simple immobilization measures and apply bandages.
  -Perform wound suturing using the appropriate knot technique and remove sutures.
  -Perform septic and aseptic dressing changes.
- ☐ Students are familiar with various non-pharmacological therapy principles for topical wound healing and can apply them according to the needs of the patient.
- ☐ None of the options

Question type: Multiple Response (MR)

4 b. Do you teach the learning objectives for wound management from the German Network for Quality Development in Nursing (DNQP) - expert standard for people with chronic wounds in your degree program? (only for nursing/nursing sciences)

- Students are able to take a wound assessment.
- Students can perform wound treatment (as prescribed by a doctor) and recommend appropriate wound dressings with justification.
- Students have management and implementation skills related to the care of people with chronic wounds.
- Students perform wound care in an evidence-based manner.
- Students are able to classify the wound and the wound environment.
- Students assess wound care.
- Students justify the wound management used in an interprofessional team.
- None of the options

Question type: Multiple Response (MR)

5. Can you please describe how you teach wound management in your classes? (When in the program, scope, main topics, teaching/learning methods)

Question type: Free text

6a. Do you teach wound management in an interprofessional setting? (e.g., nursing, medicine, pharmacy)

- Yes
- No

6b. Can you please describe how you teach wound management in an interprofessional manner? (Time during the course of study, scope, main topics, teaching/learning methodology, which courses)

Question type: Dichotomous question (yes/no question) + free text
